# Impact of COVID‐19 pandemic and the use of telemedicine on the diagnosis and treatment of tinea corporis: An experience of Southern Italy center

**DOI:** 10.1111/dth.15789

**Published:** 2022-09-06

**Authors:** Vincenzo Picone, Lucia Gallo, Gabriella Fabbrocini, Fabrizio Martora

**Affiliations:** ^1^ Section of Dermatology—Department of Clinical Medicine and Surgery University of Naples Federico II Napoli Italy

Dear Editor,

Tinea corporis has a characteristic clinical presentation with the presence of an annular, red, centrifugally developing patch with a central area of resolution and active border. It can often be accompanied by itchy symptoms.^1^ Lesions may be single or multiple and the size generally ranges from 1 to 5 cm, however larger lesions and confluence of lesions can also occur.^2^ The diagnosis of tinea corporis is clinical but can be confirmed with a potassium hydroxide (KOH) examination of skin scrapings. Direct microscopic examination by 10%–20% KOH is rapid and inexpensive and helps greatly in some situations where there is a diagnostic suspicion of more than one disease.^3^

We conducted a 2‐year data collection at our center where we evaluated the impact of covid‐19 on diagnosis and treatment of tinea corporis.

During the pandemic period caused by COVID‐19, to contain the rapid spread of the virus, the Italian government decided to introduce several measures to contain contagions such as lockdown and quarantine starting from March 10, 2020. These measures inevitably caused a longer latency between visits performed and a lower number of accesses to the mycology outpatient clinic of the University of Naples “Federico II.” Patients were referred to us by dermatologists suspecting Tinea Corporis as a diagnosis. In the following work we analyzed the impact of these restrictions on the diagnosis and therapeutic response of patients affected by Tinea Corporis, comparing the pre‐pandemic year (March 2019–March 2020) with the following year during the pandemic (March 2020–March 2021). During the year between March 2019 and March 2020, both initial and follow‐up visits were being performed at our clinic. Instead, in the pandemic year between March 2020 and March 2021, all visits of first access for suspected Tinea corporis were made at our facility, while the 2 months follow‐up visits after diagnosis were made in Telemedicine. Therapeutic response was established with the resolution of manifestations clinically objectified in the pre‐pandemic year (March 2019–March 2020). Instead in the following year, therapeutic response was assessed in telemedicine by patients' anamnestic data and by photos sent.

The first finding was the decrease in the number of visits performed (Table 1), probably because many visits were deferred due to COVID‐19 and to the restrictions implemented by the Italian government on 10 March 2020. The average age of the patients who were visited was of 40 ± 2.7 standard deviation (SD) (March 2019–March 2020) versus 45 ± 3.2 SD (March 2020–March 2021). Moreover, in the period March 2020–2021, we observed a significant decrease in percentage of mycologically confirmed positive cases of Tinea Corporis (Table 1) compared with the percentage of positive patients in the period March 2019–2020 (77.6% vs. 59.8%, *p* < 0.01). The average duration in months of Tinea Corporis was of 2 ± 1.2 SD in the pre‐pandemic year, whereas in the year during the pandemic was of 3 ± 1.8 SD. We have also identified a significant decrease of resolution of manifestations at the 2 months follow‐up visit in the period 2020–2021, compared to the previous year (90.9% vs. 66.3%, *p* < 0.01) A chi‐squared test was used to test our hypothesis.

**TABLE 1:** Epidemiological and clinical features of patients

Year	March 2019–March 2020	March 2020–March 2021
Number of visits	2324	1372
Average age (years)	40 ± 2.7 SD	45 ± 3.2 Standard Deviation SD
Positive diagnosis for tinea corporis using direct microscopic examination by 10% KOH and/or by culture examination	1804 (77.6%)	821 (59,8%)
Average duration of Tinea Corporis (months)	2 ± 1.2 SD	3 ± 1.8 SD
Resolutions of manifestations after 2 months	1641 (90.9%)	545 (66.3%)

Abbreviation: SD, standard deviation.

What emerges from this data is that the pandemic period had a significant impact on both diagnosis and responsiveness to therapies of patients affected by Tinea Corporis. In fact, patients, during the pandemic year (March 2020–March 2021), accessing with a greater latency to the first visit to our facility, had delayed diagnoses often complicated by home treatments.

We believe that the measures taken during the pandemic may have also affected the therapeutic response of patients, as it is known that dermatophytes have a marked tendency to develop resistance to antifungal agents in inveterate cases.^4^ Confirming this in our study at follow‐up visits after 2 months performed in telemedicine during March 2020–2021, more treatment failures were found comparing to the previous year. In addition, the mean duration of clinical manifestations was significantly longer in the pandemic year.

Teledermatology played a central role during the COVID‐19 pandemic, which led to a strong development of this type of service, providing ample satisfaction for both physicians and pa Although works in the literature suggest how helpful this service has been,^4^, ^5^, ^6^, ^7^, ^8^ in our experience not all dermatologic conditions can benefit equally from this type of visit.

In conclusion, it is often not easy to manage or explain a treatment through this modality, so we believe that there are still considerable difficulties for therapeutic management as our data show.

We believe that new guidelines are needed for the future to improve these services and increase the satisfaction of both patients and dermatologists.^9^

## CONFLICT OF INTEREST

The author declares that there is no conflict of interest. None of the contributing authors has any conflict of interest, including specific financial interests or relationships and affiliation relevant to the subject matter or discussed materials in the manuscript.

## FINANCIAL DISCLOSURES

None to declare. All authors equally contributed to the work.

## INFORMED CONSENT

Patient gave her informed consent for publication of her case.

## Data Availability

The data that support the findings of this study are available from the corresponding author upon reasonable request.
